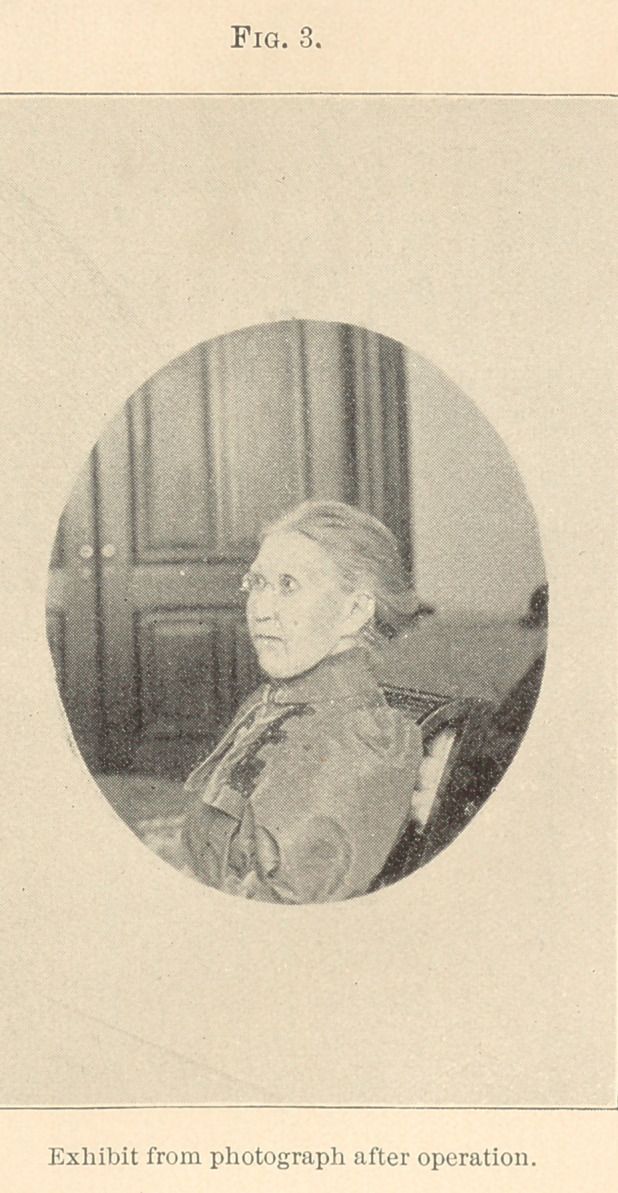# Correction of Deformities of the Oral Region

**Published:** 1893-06

**Authors:** Albert Westlake

**Affiliations:** New York


### CORRECTION OF DEFORMITIES OF THE ORAL REGION.[1]

BY ALBERT WESTLAKE, D.D.S., NEW YORK.

In October, 1892, while on a professional visit in the West, I was requested to consult with a lady in regard to a deformity of the oral region.

The history of the case is as follows: In 1870, Mrs. X., aged sixty, through the indiscretion of her husband, contracted syphilis. She first complained of soreness in the region of the nose and malar bones, and an itching and pain in the hard palate. Subsequently the turbinated, vomer nasal, and palate bones were removed. The result, after twenty years, is represented in Figs. 1, 2, and 3. As is often the result in such cases, she was addicted to morphine for a long period, but at this time, the disease having been arrested, the habit is entirely overcome.

I hesitated to attempt her case, it having been pronounced by eminent dentists in her city as beyond remedy. The only remaining three teeth on the upper arch were shells, except the right bicuspid, although the roots were firm.

The successful adaptation and use of an artificial denture would apparently depend on the support of these teeth.

She came to New York, however, and I succeeded in cleansing and filling the root-canals, and fitting gold caps over each tooth, and soldering them together, making a perfect union. They were closely fitted under the gingival margin of the gum, and, being highly polished, were comfortable and perfectly aseptic.

I next fitted an upper denture, which closed the opening in the hard palate and articulated naturally with the lower teeth.

In the adjustment of a nose I followed a plan I had heretofore used in such cases.

First taking a plaster cast of the head (and in the case of this patient being aided by an old photograph), I succeeded by a careful appreciation of the general facial contour in preparing a preliminary nose in wax. This is made as near as possible in color to the complexion, so as to secure the proper blending of shade and expression. When satisfactory to my patient and myself, I made an aluminum cast, about a line in thickness, and in weight not exceeding four pennyweights.

---

[1] Read before the New York Odontological Society, March 21, 1893.

Fig. 1.

Exhibit of model of jaw.

Fig. 2.

Exhibit from photograph before operation.

Fig. 3.

Exhibit from photograph after operation.

Over this I painted the flesh enamel, being a preparation of wax, gum-arabic, gutta-percha, etc., which resembles closely the translucency of flesh, and is not affected by ordinary thermal changes. After the nose was thus prepared and adjusted to the face, I was fortunate in securing the services of Mr. Constant Thys, the artist in charge of the Eden Musée, who imparted the life-like appearance, blending with the complexion so closely, even to the wrinkles, pores, and veins, as to challange detection.

The main difficulty was then presented in securing the nose to the face. I overcame this, after several trials, by fastening a bar of gold and platina to the inner ridge of the nose and passing it down at a carefully studied angle through the artificial denture. Various devices were tried to secure the bar after passing through the plate.

I found that although a nut and triple thread would work well when I used the forceps or a specially prepared holder, yet the patient found it exceedingly awkward. I also rejected the plan of securing the bar by a spring nut similar to those used in French clocks, fearing that if it suddenly loosened the patient would swallow it.

I finally decided and adopted—and much to the pleasure and comfort of my patient—the simple extension on the plan of a blade and spring in a pocket-knife. (I was assisted in its construction by the W. F. Ford Surgical Instrument Company.)

As the nose was placed on the face and the bar passed through the hole in the plate, she could, by simply pressing the extension, spring it tightly in a groove in the plate, and as easily remove it.

The nose when adjusted is represented in Fig. 3. The only apparent joining that can be noticed is immediately below the junction of the eyebrows, and this is not visible when the patient wears glasses.

Spectacles are not necessary to the adaptation of this nose. In masticating there is no movement of the nose.

In a recent case, presented to me by Dr. Charles McBurney, surgeon to Roosevelt Hospital, I was enabled to secure the artificial nose with the aid of gold bars and rubber attachments, but consider the additional bracing by spectacles advisable in most cases. Where special arrangement is required, I have been aided in their adjustment by Mr. W. E. Duncan, of E. B. Meyrowitz, opticians.

As shown in Fig. 2, there was a partial destruction of the soft palate.

As our authorities state, and as we well know, this organ is an important factor in the perfection of human speech. The voice, as it issues from the larynx, is modified in tone and character and interrupted in its passage by certain organs, the most important being the velum palati. This acts as a curtain, directing all the sound at times through the mouth, and again, combined or entirely, through the nasal passages, by being in firm contact with the dorsum of the tongue. The soft palate, in conjunction with the muscular wall of the pharynx, must be under active control to secure the purity of speech. A certain indistinctness of utterance follows where the soft palate is deficient or deformed (as often found after operations of staphylorraphy, when the newly-formed septum is rigid, tense, and deficient in length), or the pharyngeal walls are paralyzed or inactive.

Although the velum lost in this case is not very extensive, requiring only an obturator less in size than a pigeon's egg, yet I was unable to fit it with comfort, owing to the gagging or apparently suffocating sensation it caused. The restoration of speech to this patient is remarkable. The improvement is marked in the vowels and diphthongs, because the plate covering the aperture in the hard palate allows the resonance in the buccal cavity—where all vowels are made—and closes the abnormal communication with the nasal cavity. The physiological action of the nares and nostrils is, of course, important for the production of purity in speech, and and as this nose is mechanical, there remains imperfect sounds which practice will hardly overcome.

The patient, at my suggestion has commenced lessons in elocution. I have advised the study of the French or some foreign language, mainly to divert the mind from the forced habit of speaking her own vernacular badly.

The faculty of fairly perfect speech is, in most cases, more easily acquired in accidental lesions than in congenital clefts. A well-known and recent authority on the deformities of the oral region says, "The attempt at the replacement of a nose may be regarded as exceptional for the reason that it is so conspicuous an organ that the appearance is of more consequence than utility. There is no organ in the entire economy the loss of which creates so hideous a deformity as the nose, nor is the successful prosthetic restoration of any organ so difficult.

"Artificial teeth, eyes, legs, hands, arms, etc., are all made so skilfully as to escape detection, but artificial noses, never. The best of them, however artistic or appropriate in form, are but poor imitations of adjacent living tissue."

This complete assertion was in mind when my first attempt in this specialty was commenced, but as a result of careful investigation and study, and the enamel preparation used, together with the artist's treatment, and the light-weight metal, has enabled me to produce results which have been pronounced by some of our eminent surgeons as remarkable.

It was my desire to present several other cases to you this evening, but I have not been able to complete them in time.

This plaster model shows a remarkable congenital deformity of the ear. The patient is a splendidly-developed lad of twelve years. The ear on the right side is beautifully formed, as you will observe by this other plaster model.

Several weeks ago, Dr. Charles McBurney made a very skilful operation, opening through this stub close to the skull, giving me a slit half an inch long and very strong, to which I shall attach the ear made of similar material to the nose.

I have several other cases, including replacement of nose and malar bones, of entire bridge of nose and of inferior maxillary, which I shall be pleased to present to the profession if they so desire.

## Figures and Tables

**Fig. 1. f1:**
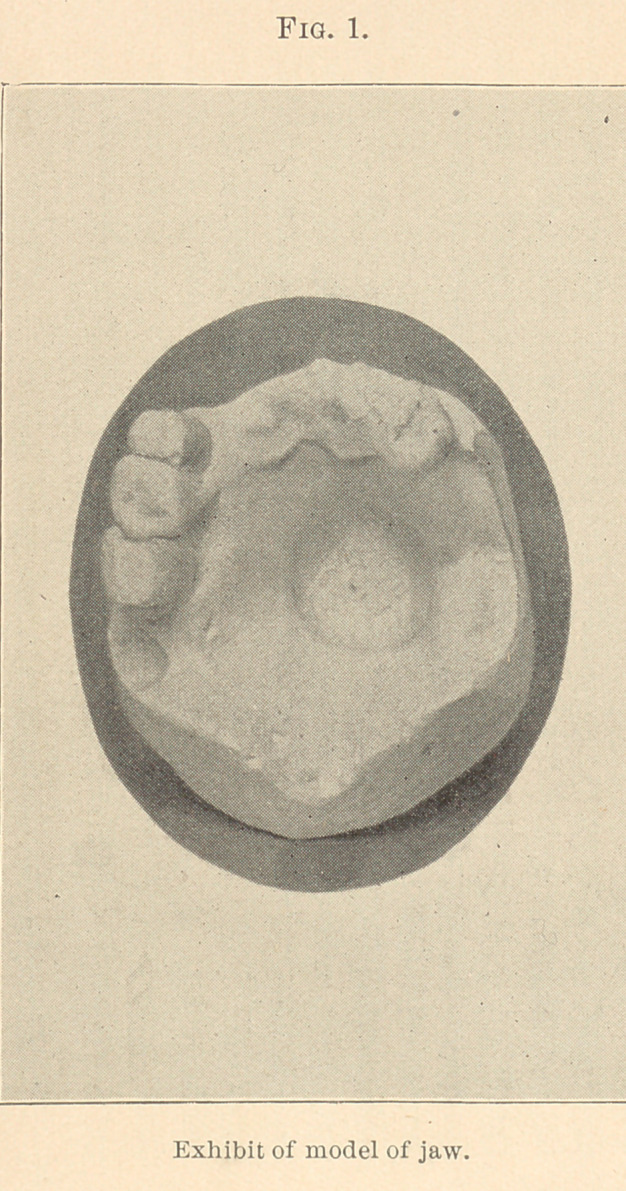


**Fig. 2. f2:**
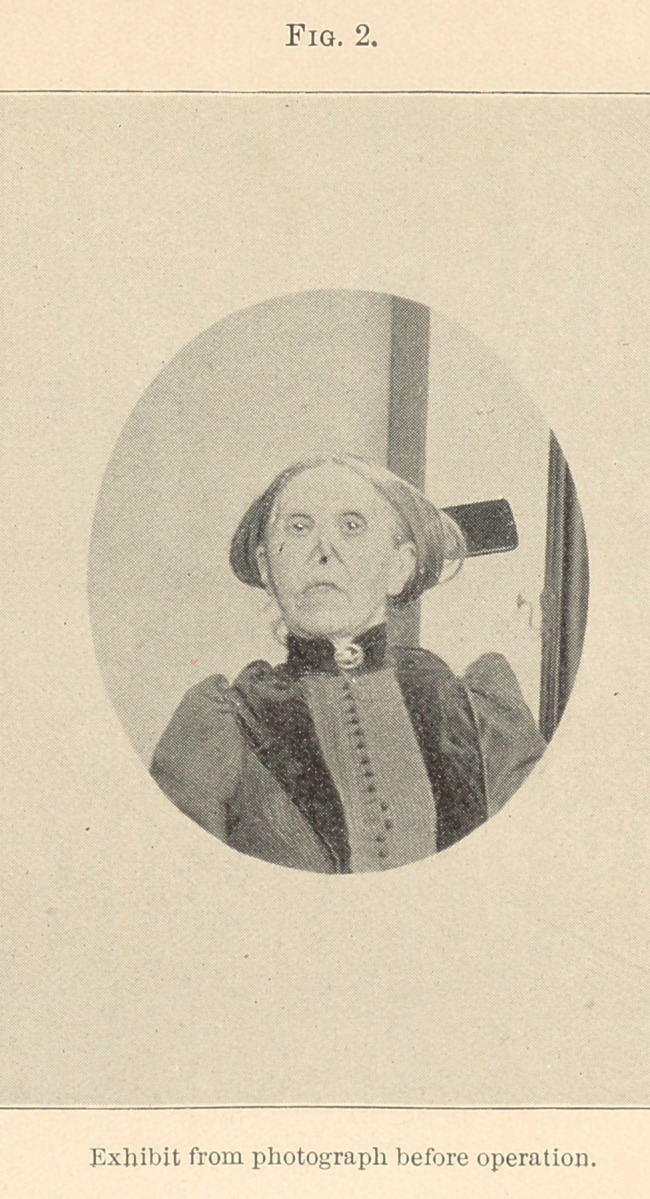


**Fig. 3. f3:**